# Developing a preliminary ‘never event’ list for general practice using consensus-building methods

**DOI:** 10.3399/bjgp14X677536

**Published:** 2014-02-24

**Authors:** Carl de Wet, Catherine O’Donnell, Paul Bowie

**Affiliations:** General Practice & Primary Care, Institute of Health & Wellbeing, College of Medical, Veterinary and Life Science, University of Glasgow, Glasgow.; Professor of primary care R&D, General Practice & Primary Care, Institute of Health & Wellbeing, College of Medical, Veterinary and Life Science, University of Glasgow, Glasgow.; Safety & Improvement, Postgraduate GP Education, NHS Education for Scotland, Glasgow.

**Keywords:** general practice, never events, patient safety, primary care

## Abstract

**Background:**

The ‘never event’ concept has been implemented in many acute hospital settings to help prevent serious patient safety incidents. Benefits include increasing awareness of highly important patient safety risks among the healthcare workforce, promoting proactive implementation of preventive measures, and facilitating incident reporting.

**Aim:**

To develop a preliminary list of never events for general practice.

**Design and setting:**

Application of a range of consensus-building methods in Scottish and UK general practices.

**Method:**

A total of 345 general practice team members suggested potential never events. Next, ‘informed’ staff (*n* =15) developed criteria for defining never events and applied the criteria to create a list of candidate never events. Finally, UK primary care patient safety ‘experts’ (*n* = 17) reviewed, refined, and validated a preliminary list via a modified Delphi group and by completing a content validity index exercise.

**Results:**

There were 721 written suggestions received as potential never events. Thematic categorisation reduced this to 38. Five criteria specific to general practice were developed and applied to produce 11 candidate never events. The expert group endorsed a preliminary list of 10 items with a content validity index (CVI) score of >80%.

**Conclusion:**

A preliminary list of never events was developed for general practice through practitioner experience and consensus-building methods. This is an important first step to determine the potential value of the never event concept in this setting. It is now intended to undertake further testing of this preliminary list to assess its acceptability, feasibility, and potential usefulness as a safety improvement intervention.

## INTRODUCTION

Patient safety in health care is now a global concern because of mounting evidence, particularly in acute hospital settings, that patients are unintentionally but frequently harmed in situations often judged to have been preventable. Such events are difficult, sometimes harrowing, and when extreme can result in organisational change and widespread media reporting, such as observed during and after the Francis Inquiry into the deaths at the Mid Staffordshire Hospitals NHS Trust.^1^,^2^ In response, many countries, including the UK, have devised and implemented national improvement strategies to reduce avoidable harm,^3^–^5^ including the introduction of policies to help prevent ‘never event’ occurrences.^6^

The current definition of a never event is ‘a serious, largely preventable patient safety incident that should not occur if the available preventable measures were implemented by healthcare workers’.^7^ An unambiguous example of a never event in the acute hospital context is performing a surgical procedure on the wrong limb. The rationale for devising and implementing lists of never events in health care, therefore, is to mitigate or eliminate the risks associated with these types of serious but preventable occurrences.

Never events were conceived in the US after legislation in 2006 which constrained hospitals’ ability to financially charge a medical insurer or patient for eight selected ‘higher-paid diagnosis-related’ groups if certain clinical complications were to occur.^8^ These clinical groups were selected from the list of ‘serious reportable events’ and became known as never events: a phrase coined because of its ‘extra psychological charge’.^8^,^9^

Never event lists have now been developed and implemented for acute hospital settings in many national healthcare systems, including the NHS in England & Wales,^6^ and increasingly for specific settings and clinical disciplines.^10^–^16^ Never event policies are also in place for some community-based settings, such as home care agencies^17^ and community nursing.^18^,^19^

This attention to never events confers at least four potential benefits. First, they can increase awareness of highly important patient safety issues among the healthcare workforce. Second, preventive measures can be implemented by frontline organisations and teams, thereby proactively improving the safety and quality of health care. Third, local healthcare organisations can alert frontline care workers and teams of never event policies and work with them to put in place preventive strategies, for example by the promotion and introduction of surgical ‘time outs’ and checklists to help prevent wrong site surgery.^10^ Finally, there is increased accountability to patients and the public in acknowledging and dealing with serious patient safety incidents.

To date, most of the work on patient safety has focused on hospital care. The reason for this is that it reflects the dominance of hospital-based care in many health systems and is the result of a perception that this is where most serious incidents occur.^20^–^22^ However, there is now a growing recognition of the need for patient safety to be at the forefront of practice in primary care and general practice.^3^,^4^ However, it is acknowledged that this is a relatively new approach for many general practice teams and organisations and that there are gaps between actual and desired safety-critical practices.^23^–^25^ Following the strategies taken in acute care, one approach will be to develop a preliminary list of never events for the UK general practice setting. Only by having such a list, can we start to test its utility and inform the discussion about patient safety in general practice. However, such an approach should be grounded in the daily experience of general practice. The aim of this study, therefore, was to develop such a list, bringing together the views and experience of frontline primary care clinicians and managers, as well as experts in the field of patient safety. In addition, the study sought to develop and apply a set of criteria which could be used to judge the validity of potential never events for any primary care setting.

**How this fits in**

‘Never event’ lists have been used in many secondary care settings in the UK and internationally as part of efforts to improve patient safety. Patient safety research in general practice is limited but growing, with increasing evidence for the occurrence of avoidable patient harm. A never event list provides a potential new approach to engage frontline staff in explicitly considering and acting on a range of safety-critical issues which may cause avoidable harm to patients in general practice. A preliminary list of never events for general practice was developed through practitioner experience and consensus-building methods. Definitional criteria were also developed that are necessary to help identify never events in this setting. This preliminary list is an important first step to determine the potential value of the never event approach as a safety improvement intervention in general practice.

## METHOD

### Study design

A range of consensus-building methods were used with frontline general practice staff and patient safety experts, including qualitative generation of potential never events, a consensus-building workshop, modified Delphi exercise, and a content validation exercise.

### Data collection and analysis

Data were collected, analysed, and integrated in three consecutive stages.^26^ These are described in more detail below.

#### Stage 1: Identification of potential never events

A rapid scoping review was undertaken of the existing international literature to identify key threats to patient safety in primary care settings, with particular emphasis on previously published comprehensive evidence summaries.^23^,^27^,^28^ The search strategy included searching and screening internationally published literature using relevant electronic databases, including Medline, Embase, Cinahl, and Psych-Info (1990 onwards). Sources of relevant information in the grey literature were also searched and screened, for example conference abstracts and policy documents for reported incidents that fitted the description of never events given in the introduction.

In addition, the concept of never events was introduced to GPs, practice nurses, and practice managers at eight regional and national educational workshops, meetings, and conferences over a 12-month period from November 2011 to November 2012 in Scotland. These included the national meeting of Scottish departments of general practice and primary care, the Scottish School of Primary Care conference, the Medical Appraisal National Conference, and the North GP Trainers’ Annual Conference.

The choice of workshops, meetings, and conferences was pragmatic, and typically the result of one of the authors being invited to attend them as a presenter. The never event concept was introduced opportunistically and all attendees were invited to participate. The only selection criteria were that they had a role in primary care and agreed to participate. However, this means that there was less control over who chose to attend and who did not. Participants were asked to discuss the concept in small groups and self-reflection on past experiences of patient safety incidents was encouraged. The participants were then invited to suggest potential never events, which were collected in writing using a simple proforma. The proforma also included the definition of a never event^7^ and a number of examples from secondary care settings.

All the data from the completed proformas and findings from the literature search were combined into a list of potential never events. The list was reviewed; duplicate and similarly related patient safety incidents were merged, rephrased, or removed; obviously humorous suggestions were also removed. Potential never events were then grouped into main categories according to the emergent themes jointly identified.

#### Stage 2: Development and application of never event criteria

A multidisciplinary group of clinical, managerial, and administrative primary care staff with leadership roles in patient safety initiatives from across Scotland was invited to attend a full-day never event workshop in Glasgow in January 2013. Potential participants were identified based on previous collaboration with the authors, through networking, or because of their roles at a national or health board level in the NHS, in NHS Education for Scotland (NES) or NHS Quality Improvement Scotland (QIS), and NHS health boards.

The first aim of the workshop was to develop and agree criteria specific to the general practice setting which could be used to identify a never event. The second aim was to apply these criteria to the list of potential never events created during stage 1. The aim of this stage was to ensure that potentially ambiguous, impractical, or highly contentious incidents were excluded, and that the retained items were perceived as both dangerously harmful and relevant to a general practice setting.

During the first part of the workshop the delegates were presented with background information about the never event concept and a worksheet to complete during the morning session. The worksheet is available as a supplementary file and includes a copy of the National Patient Safety Agency’s (NPSA) five criteria for defining never events in acute hospital and mental health settings.^29^ In addition, seven potential criteria specific for general practice, which were informed by and adapted from the NPSA approach coupled with previous professional and research experience, were proposed and included. The delegates considered each criterion in turn and discussed its relevance and clarity in an open forum facilitated by one of the authors. Each criterion was considered, refined, and adapted until consensus was reached on its clarity and relevance for general practice. Only once every single group member was satisfied as to the validity of the criterion, as indicated by verbal consent, a show of hands, and by completing the worksheet, was it included in the final set.

The group then applied these definitional criteria to the list of potential never events. Each suggested incident was discussed in small groups of five or six delegates and again in the main forum. The authors facilitated these discussions, made contemporary notes and clarified and edited items according to group feedback. Potential never events were only included on the candidate list if there was unanimous group consensus that each event fully satisfied all of the criteria. This process comprised robust debates between participants over interpretations and application of the agreed criteria for inclusion/exclusion of individual items before final agreements could be reached.

#### Stage 3: Content validation by an expert group

Patient safety ‘experts’ in UK primary care were identified to help refine and validate the list of candidate never events. They were accorded ‘expertise’ based on relevant peer reviewed journal publications and/or NHS occupation (for example, Patient Safety Manager). To identify clinicians/researchers who had published on patient safety in primary care issues, online website searches were conducted of the following journals: *BMJ Q&S, QPC, IJQHC, BMC Family Practice, BJGP, Family Practice,* and *Implementation Science*. Also, NHS clinicians and safety managers were selected who held senior leadership roles in national or regional patient safety initiatives in primary care. The experts were recruited by email and invited to participate in a modified Delphi exercise and to complete a content validity exercise.

In the first round, the experts were asked to grade each candidate never event using a 4-point rating scale. They were asked to: ‘Please indicate if the following statement meets the pre-defined criteria for a ‘never event’, where:

1 = not at all;
2 = it may be but only if reworded substantially;
3 = it fulfils the criteria for a never event but I suggest the following minor alteration(s); and
4 = it is a never event and should be included in the final list.

In addition, the experts were invited to edit each never event to enhance its clarity, raise any concerns, suggest additional never events, and provide general feedback. The expert group’s feedback was then collated and used to edit the candidate never events.

In the second and final Delphi round, the revised list of candidate never events and the group’s anonymised scores were forwarded to each expert. They were again asked to edit each never event if they deemed this necessary and specifically to reconsider their previous ratings, leading to a small number of minor revisions. Finally, the revised never events were included in the final list if they had been rated as a ‘3’ or ‘4’ by a minimum of 80% of experts: the content validity index (CVI).

## RESULTS

### Potential never events

A total of 345 primary care team members (243 GPs, 23 practice nurses, and 79 practice managers) provided 721 written suggestions of potential never events. All of the suggestions are available in a supplementary file. The suggestions largely correlated with the findings from the literature review, so that the initial list of 721 potential never events remained unchanged. This list was reviewed by the authors and consensus reached on reducing the 721 initial suggestions to 38 potential never events (Table 1).

**Table 1.** List of potential never events and main themes

**Section A: Mistaken patient identity**
1. The wrong action is taken, or the right action is taken but for the wrong patient, for example, referral, clinical entry, prescription, acting on a test result, or drug administration.

**Section B: Acts of omission**
2. An agreed referral is not made.
3. Transport (ambulance) is not arranged while admitting a patient as an emergency.
4. Discharging patients without advance notification of practice, district nurses, or making necessary arrangements.
5. Not carrying out an agreed house visit.

**Section C: Investigations**
6. An abnormal investigation result is not received by the practice that requested it.
7. An abnormal test result is received by a practice but not considered for action, or the considered action is not performed.

**Section D: Medication (prescribing, dispensing, administration, monitoring)**
8. The ‘wrong’ drug is prescribed, dispensed, or given.
9. Drugs are prescribed at the request of non-practice clinicians and from other healthcare settings without clear, complete, and written requests.
10. Prescribing medication when known, absolute contraindications exist:
10a.Prescribing teratogenic drugs to a patient known to be pregnant.
10b.Specific previous incidents, for example, combined oral contraceptive after previous confirmed DVT/PE.
10c.Specific medical conditions (metformin, nitrofurantoin or NSAIDs in renal failure (or eGFR <30); beta-blockers for asthmatics; oestrogen only HRT for women with intact uterus).
10d.Previous allergic reaction to the drug.
11. Prescribing two drugs with known and potential serious interaction together.
12. Prescribing or giving the wrong dose of medication. Specifically, prescribing doses higher than the maximum recommended in the *BNF*.
13. Making changes to medication (dose, new, discontinue) without informing the patient or patient representative and documenting the change and rationale.
14. Prescribing ‘high risk’ medication without ensuring adequate monitoring took place and results were satisfactory.
15. Dispensing medication or providing a prescription to anyone other than the patient or patient representative.
16. Giving the right drug via the wrong route or at the wrong site.
17. Failure to reconcile medication after receiving hospital discharge documentation.

**Section E: Medico-legal and ethical incidents**
18. Non-clinical team members should not perform clinical tasks.
19. Physical assault of patients or healthcare workers.
20. ‘Ignoring’ a patient’s living will.
21. Breaching patient confidentiality.
22. A practice team member works while intoxicated.
23. ‘Losing’ controlled drugs.
24. Accessing patient records for purposes other than delivery of care.
25. Performing invasive or intimate procedures without offering a chaperone.

**Section F: Clinical management**
26. Omission of certain, specific clinical actions in given scenarios are ‘never events’.
26a.Prescribing repeated courses of antibiotics without a clinical assessment.
26b. Not examining a febrile child.
26c.Not obtaining and recording a blood pressure reading for patients presenting with acute-onset headache.
26d.Not recording a peak-flow measure in patients with asthma presenting with an acute exacerbation.
26e.Not referring a patient presenting with and treated for anaphylaxis to secondary care for observation.
26f.Not referring a child suspected to have non-accidental injuries urgently.
26g.Performing a speculum examination in patients >36/40 pregnant and presenting with PV bleeding.
27. A patient suffers ‘severe burns’ from cryotherapy.
28. Using non-sterile equipment.
29. Performing a cervical smear without visualising the cervical os.

**Section G: Practice systems**
30. A practice does not have an up-to-date and secure backup of their data.
31. Medical waste and hazardous substances discarded in an inappropriate manner.
32. If equipment is not in working order, maintained, available, or checked regularly.
33. Inappropriate triage or refusal of access.
34. Sending correspondence to a deceased patient.
35. Patients should never be unsupervised (left alone) inside the practice.
36. A death in the practice.

**Section H: Teamwork and communication**
37. A new member of staff is not made aware of the known ‘high risk’ status of a patient before a consultation.
38. Medical trainees are not provided with adequate supervision.

There were three reasons for the high attrition of suggestions:

- some potential never events included descriptions of specific incidents;
- some suggestions were generated during small group discussions, increasing the degree of duplication; and
- the authors excluded suggestions judged to be too subjective, or that did not concern the general practice setting or the safety of patients, for example ‘giving the wrong baby to a mother’ and ‘a patient should never be regarded as wasting time’.

It is acknowledged that some of the items on this list may appear imprecise or lacking in context. This is intentional, as the study retained any item where there was even a modicum of doubt as to their potential relevance and to reduce selection bias. In this way, the multidisciplinary group was provided a potential list representative of frontline staff’s suggestions, but which was still feasible for them to review in the available time.

### Never event criteria and candidate never events

The characteristics of the 15 Scottish delegates who attended the never event workshop are shown in Table 2. Their mean reported work experience in primary care settings was 20.7 years (range 5–34 years), 10/15 (67%) were female, and most indicated they had at least two different professional roles, for example GP appraiser and educator, or practice manager and locality coordinator. Participants came from both urban and predominantly rural NHS Boards; three were regional or national representatives. However, there was no discernible difference in response between urban and rural participants.

**Table 2.** Characteristics of delegates (*n* = 15) who attended the never event workshop to agree never event criteria and candidate never events

**Profession**	**Role**	**Years of professional experience**	**Sex**
General practice	GP Appraiser and Educator	15	M
General practice	GP Associate Clinical Director	5	M
General practice	GP Educator	20	M
Practice manager	Practice Manager	15	M
Practice nurse	Education Adviser	15	F
Clinical risk	Clinical Risk Coordinator	15	F
Pharmacy	Pharmacist; Assistant Director of Pharmacy Education	25	F
Practice nurse	Education Adviser	25	F
Pharmacy	Lead Clinical Pharmacist	14	F
General practice	National Clinical Lead for Patient Safety	22	M
General practice	Director of Postgraduate GP Education	29	F
Practice nurse	Education Adviser	23	F
Clinical risk	Clinical Risk Coordinator	33	F
General practice	Practice manager, Locality Coordinator	21	F
Practice nurse	National Coordinator for General Practice Nursing	34	F

The never event definitional criteria generated are shown in Box 1. The criteria were judged to be suitable for all general practice settings, whether urban or rural. Application of the criteria to the potential never events generated in Stage 1 and feedback from the group resulted in a list of 11 candidate never events.

**Box 1. Never event criteria for general practice settings**

**A never event ..**

1. Is known to cause severe harm to a patient, or has the potential to do so AND
2. Is preventable by the healthcare professional, team, or organisation AND
3. Can be clearly and precisely defined AND
4. Can be detected AND
5. Is not the result of an unlawful act

### Refinement and validation of final never events by an expert group

Of the 29 experts identified and invited, 17 (59%) agreed to participate and completed the first modified Delphi round. Two of the 17 had previously participated in the multidisciplinary group in Stage 2 of the development process. Fourteen of the 17 (82%) amended their original ratings during the second round. The expert group endorsed 10 never events with CVI scores of >80%. The final never events and their CVI scores (as a percentage) overall and the ratings of each expert reviewer are shown in Table 3. A single candidate never event, ‘Prescribing Disease-Modifying Antirheumatic Drugs (DMARDs) if the monitoring specified in the practice’s near-patient testing protocol had not been performed or discussed with the patient’, was omitted from the final list because expert ratings were below the 80% threshold.

**Table 3.** Preliminary list of never events for UK general medical practice with individual and combined Content Validity Index (CVI) scores

**Never event**	**Overall CVI score, %^a^**	**Expert reviewers (*n* = 17)**																
		**A**	**B**	**C**	**D**	**E**	**F**	**G**	**H**	**I**	**J**	**K**	**L**	**M**	**N**	**O**	**P**	**Q**
1 Prescribing a drug to a patient that is recorded in the practice system as having previously caused her/him a severe adverse reaction	100	4	3	4	4	4	4	4	4	3	4	4	3	4	4	4	4	3
2 A planned referral of a patient, prompted by clinical suspicion of cancer, is not sent	100	4	4	3	4	3	4	4	4	4	4	4	3	4	4	4	3	4
3 Prescribing a teratogenic drug to a patient known to be pregnant (unless initiated by a clinical specialist)	100	4	4	4	4	3	4	4	4	3	4	4	4	4	4	4	4	3
4 Emergency transport is not discussed or arranged when admitting a patient as an emergency	94	3	2	4	4	4	4	4	3	4	4	4	3	4	4	4	3	4
5 An abnormal investigation result is received by a practice but is not reviewed by a clinician	94	3	3	3	4	3	3	4	3	2	4	4	4	4	4	3	3	4
6 Prescribing aspirin for a patient <12 years old (unless recommended by a specialist for specific clinical conditions for example, Kawasaki’s disease)	94	4	4	3	4	1	4	4	4	4	4	4	4	4	3	4	4	3
7 Prescribing systemic oestrogen-only hormone replacement therapy for a patient with an intact uterus	94	4	4	4	4	4	4	4	1	4	4	4	4	4	3	4	4	4
8 Prescribing methotrexate daily rather than weekly (unless initiated by a specialist for a specific clinical condition, for example, leukaemia)	88	4	4	4	4	4	4	4	2	4	4	4	3	4	4	4	2	4
9 A needle-stick injury caused by a failure to dispose of ‘sharps’ in compliance with national guidance and regulations	88	4	1	4	4	4	4	4	4	4	4	4	2	4	4	3	4	4
10 Adrenaline (or equivalent) is NOT available when clinically indicated for a medical emergency in the practice or GP home visit	88	4	4	4	4	2	4	4	3	4	1	4	4	4	3	4	4	4

Rating scale: 1 = not at all; 2 = it may be but only if reworded substantially; 3 = it fulfills the criteria for a never event but I suggest the following minor alteration(s); 4 = it is a never event and should be included in the final list.

a Expert responders who rated the candidate never event ‘3’ or ‘4’.

## DISCUSSION

### Summary

A preliminary list of never events was developed for use in the general practice setting. To the study’s knowledge, this is the first attempt to produce such a list for this setting and, although generated in the UK, the items identified are generic and have international relevance. Prescribing issues were a strong contributor to the list, in keeping with the identification of medication-related errors as a considerable threat to patient safety in general practice.^30^,^31^ Although the current incidence of these specific never events and their likelihood of occurrence are currently unknown, all have the potential for catastrophic consequences for some patients and any systematic approach to reduce their occurrence must be valuable. A second study outcome was development and agreement on definitional criteria specific to general practice, which may help to identify and confirm further never events in international primary care settings.

### Strengths and limitations

The preliminary list of never events was informed by the work-based experience of general practice team members with different roles across Scotland. However, the sample did not include patients and was unintentionally weighted to the GP staff group, as a result of workshops being generally linked to GP meetings. This reflects the increased likelihood that the authors, two of whom are GP educational researchers, were invited to conferences more likely to be attended by GPs, rather than to a lack of willingness of particular staff groups to be involved.

Very high duplication was found in the suggested never events, which may reflect a shared awareness of the more common and serious patient safety incidents among this group. Given the inherent subjectivity and emotive potential of the never event concept, consensus-building methods were selected and two different groups of experts were recruited to help refine and validate the final list in two stages. However, despite all best intentions to ensure true consensus, it may not have been possible to eliminate all bias. In addition, there may be other never events that were not considered and the final selected never events may not be acceptable to some, although the study consider this to be a preliminary list.

Criterion 5 refers to ‘unlawful acts’, which may include, for example, accessing medical records without a clinical reason, misappropriating controlled drugs, ignoring a patient’s living will, or working while intoxicated. It is suspected that some readers will initially be surprised by the group’s unanimous decision (after a lengthy debate) that these types of incidents are not appropriate for a never event list. Although these incidents are clearly undesirable and should ideally ‘never’ happen, the study argue for their exclusion from a preliminary list of *preventable* never events for the following three reasons, using the example of ‘working while intoxicated’:

- unlawful incidents, such as this one, are violations (*deliberate* actions that are inconsistent with rules or recommended practice that should be familiar to a healthcare worker) rather than human or clinical error (the *unintentional* result of choosing the wrong plan to achieve an aim, or not initiating or completing the right plan as intended);
- never events should, by definition, be preventable in every single instance by every organisation. Although organisations may do much to detect and support healthcare workers with substance abuse problems, they cannot influence every individual’s decision; and
- the main benefits of a never event policy are reporting, analysing, and learning from an incident, to safeguard against its future occurrence. There are already existing organisational policies and procedures to deal appropriately with these types of incidents.

### Comparison with existing literature

There is currently a paucity of evidence relating to never events in primary care. As outlined in the introduction, the Department of Health’s never event framework is heavily weighted to hospital-based care. For example, wrong limb surgery is an event that is only likely to occur in hospital, whereas the lack of adrenaline (or equivalent) in the practice or during a home visit in this preliminary list is a general practice-based event. Nevertheless, evidence is now emerging that never event policy implementation is associated with improved care quality and safety in selected clinical settings.^32^–^34^ However, at least three main concerns about never event policies remain unresolved.

The first is that some never events included on current lists may not be preventable in every instance despite the best efforts, intentions, and adherence to clinical guidelines for healthcare workers and organisations.^35^–^41^ A second concern pertains to the proliferation and ‘broadening’ of never events.^42^,^43^ For example, the number of never events in the English policy has increased from eight to 25 since 2009. The potential risk is that the core essence of the never event concept as a means of focusing attention on relatively rare and serious patient safety incidents may be diluted in this process. The third concern is that never event policies may have unintended and unwanted consequences, including:

- ‘overzealous’ application of the never event approach may reduce tertiary centres’ incentive to treat high-risk patients;^44^
- ‘punitive’ policies reduce the quality and frequency of incident reporting and may lead to organisations providing incomplete data or adopting alternative metrics favouring positive outcomes;^45^,^46^
- as patient safety incidents are only considered never events if patients develop them during their healthcare journey, there is an imperative to indiscriminately screen for venous thromboembolism organisms associated with healthcare acquired infections, and any other potential ‘never event’ at first presentation, irrespective of clinical presentation;^47^
- efforts to prevent never events may divert resources, lead to ‘improvement fatigue’ among the healthcare work force, and have high opportunity costs given the relative rarity of some incidents;^8^ and
- never event lists may become synonymous with medical negligence.^48^

This seeming tension between ‘worthy goals’ and ‘overzealous application’ requires careful consideration to agree an acceptable compromise,^39^,^42^ and should also be considered in relation to the primary care setting.

### Implications for research and practice

Most never event policies have four main requirements:

- mandatory reporting of a specified incident when it occurs;
- a rigorous, organisational-level investigation to determine why the incident occurred and to identify associated risk factors;
- a responsibility to act on the findings and initiate changes to prevent a recurrence; and
- an apology to the patient concerned if appropriate.

There are a number of important challenges which will have to be considered and overcome first if these requirements are to be met in general practice. For example, how do you enforce mandatory reporting in general practice settings where engagement in voluntary incident reporting systems is minimal and inconsistent?^49^ A second challenge is whether the specified never events can be detected reliably by every practice. The authors have recently identified the work required in a practice to identify adverse events and, unsurprisingly, the rarer the event, the greater the number of patient records to be reviewed to identify such events.^50^ Other challenges include determining who should be responsible for implementation of such a policy and how this would be resourced, promoted, and prioritised.

One of the agreed definitional criteria is that a never event in general practice is preventable through implementation of existing interventions available to all at-risk clinicians and teams. There is also emerging evidence of mitigation or reduction of patient safety incidents in some of the safety-critical areas of health care from which never events were selected, for example prescribing issues^51^ and managing investigation results.^52^,^53^ However, it is currently unclear whether all of the never events in the list are truly preventable, or which of the available interventions will be most acceptable, feasible, and useful to frontline staff. Such issues require further exploration and testing in general practice. Therefore, the study intends to undertake further testing of the preliminary never event list to assess its acceptability, feasibility, and potential usefulness as a safety improvement intervention.
